# A Case Report of Hematogenous Osteomyelitis of the Manubrium Caused by Seeding from a Colovesicular Fistula

**DOI:** 10.5811/cpcem.6591

**Published:** 2024-06-03

**Authors:** Celina Wong, Tammy Phan, Emmelyn Samones, Sharmin Kalam

**Affiliations:** Loma Linda University Medical Center, Department of Emergency Medicine, Loma Linda, California

**Keywords:** *hematogenous osteomyelitis*, *sternal osteomyelitis*, *discitis*, *colovesicular fistula*, *Klebsiella pneumoniae*, *case report*

## Abstract

**Introduction:**

Osteomyelitis can occur at various osseous locations and commonly presents in the emergency department (ED). The incidence of osteomyelitis is 21.8 cases per 100,000 persons annually.^1^ Hematogenous osteomyelitis typically occurs in the vertebrae; however, it may seldomly occur in the manubrium. Hematogenous osteomyelitis can be seen in patients with complicated thoracic surgery, radiation, fracture, diabetes, immunosuppression, steroid therapy, and malnutrition.^2^ Because signs and symptoms of osteomyelitis may be nonspecific, clinicians must have high suspicion based on history and physical. Workup should include identifying the source, imaging, and surgical cultures.

**Case Report:**

A 60-year-old male with hypertension and diabetes presented with atraumatic right shoulder and chest pain. The patient presented twice to the ED for clavicle pain five days prior. Computed tomography (CT) of the chest detected osseous infection of the manubrium and upper sternum, right clavicle, and mediastinal phlegmon. A CT of the abdomen and pelvis revealed osteomyelitis and discitis of the 12^th^ thoracic and first lumbar vertebral body with gas at the psoas muscle, as well as sigmoid diverticulitis with colovesicular fistula. The patient was started on broad spectrum antibiotics and 1,500 milliliters of lactated Ringer’s in the ED. After evaluation by cardiothoracic surgery, the patient was taken to the operating room for neck exploration, incision/drainage, manubriectomy, and right sternoclavicular joint resection. Surgical, blood, urine, and respiratory cultures grew *Klebsiella pneumoniae*. After a 34-day hospital course, the patient was discharged on two weeks of oral levofloxacin and follow-up appointments with cardiothoracic surgery and infectious disease. The patient had good prognosis and recovery.

**Conclusion:**

Hematogenous osteomyelitis to the manubrium is rare and may present with only chest pain. It is important to consider other sources that seed in the manubrium and imaging to evaluate multisite infection. Treatment should include intravenous antibiotics and/or surgical intervention for debridement with washout or manubriectomy.

Population Health Research Capsule What do we already know about this clinical entity?

*Hematogenous osteomyelitis is a rare form of osteomyelitis that occurs when bacteria spreads to the bone through the bloodstream.*

What makes this presentation of disease reportable?

*We report a rare location of the hematogenous spread to the manubrium with an even more uncommon treatment course.*

What is the major learning point?

*This case teaches the importance of an extensive workup to identify the primary source of hematogenous osteomyelitis when found in rare locations.*

How might this improve emergency medicine practice?

*Emergency physicians should be aware of hematogenous osteomyelitis and how extensive workup will aid in evaluating this disease process.*

## INTRODUCTION

Osteomyelitis is defined as infection and inflammation of bone with multiple types that require different interventions and management. Osteomyelitis develops by three mechanisms: bacteremia leading to hematogenous spread to the bone; contiguous spread from adjacent soft tissue to the bone; or direct inoculation into the bone.^3^ The rarest type of osteomyelitis is that of hematogenous spread.^4^ The most common location of osteomyelitis is the vertebrae. Additionally, osteomyelitis of the manubrium (outside of thoracic surgery- related complications) is even more uncommon with very few case reports published; it can either lead to or be a result of hematogenous seeding.^5^ Typically, these patients will have multiple risk factors such as diabetes or immunodeficiency that predispose them to infection. Treatments vary from intravenous (IV) antibiotics alone to the addition of surgery. This case of manubrium osteomyelitis is unique in that it was the result of hematogenous spread; a manubriectomy was necessary in addition to long-term IV and oral antibiotics.

## CASE REPORT

A 60-year-old male with a past medical history significant for hypertension and diabetes mellitus presented to the emergency department (ED) with right-sided shoulder and chest pain. The patient initially presented five days prior with complaints of a three-day history of right clavicle pain. There was no reported direct trauma, but the patient noticed the pain started after he was catching boxes at work. A chest radiograph and dedicated right clavicle radiograph was ordered at the time, and both imaging resulted as normal. The patient was then discharged and diagnosed with a musculoskeletal strain. Five days later, the patient presented to the same ED, now with associated chest pain, fevers, and shortness of breath. Between the two presentations, the patient was seen at another facility, where computed tomography (CT) revealed osteomyelitis of the sternoclavicular joint and manubrium. According to the patient, the CT also mentioned colonic fistula and because that facility did not have a cardiothoracic specialist available, transfer arrangements were attempted but were unsuccessful. Therefore, the patient decided to leave against medical advice and present to the ED directly. The patient denied any IV drug use, tobacco use, or history of autoimmune disorders.

**Image 1.**
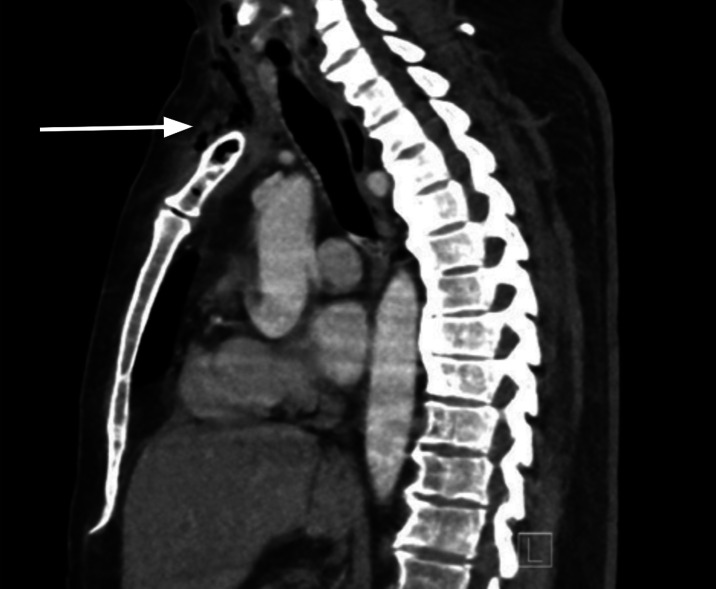
A sagittal view of computed tomography of the chest, revealing osseous infection of manubrium with surrounding gas (arrow) extending into the neck.

**Image 2.**
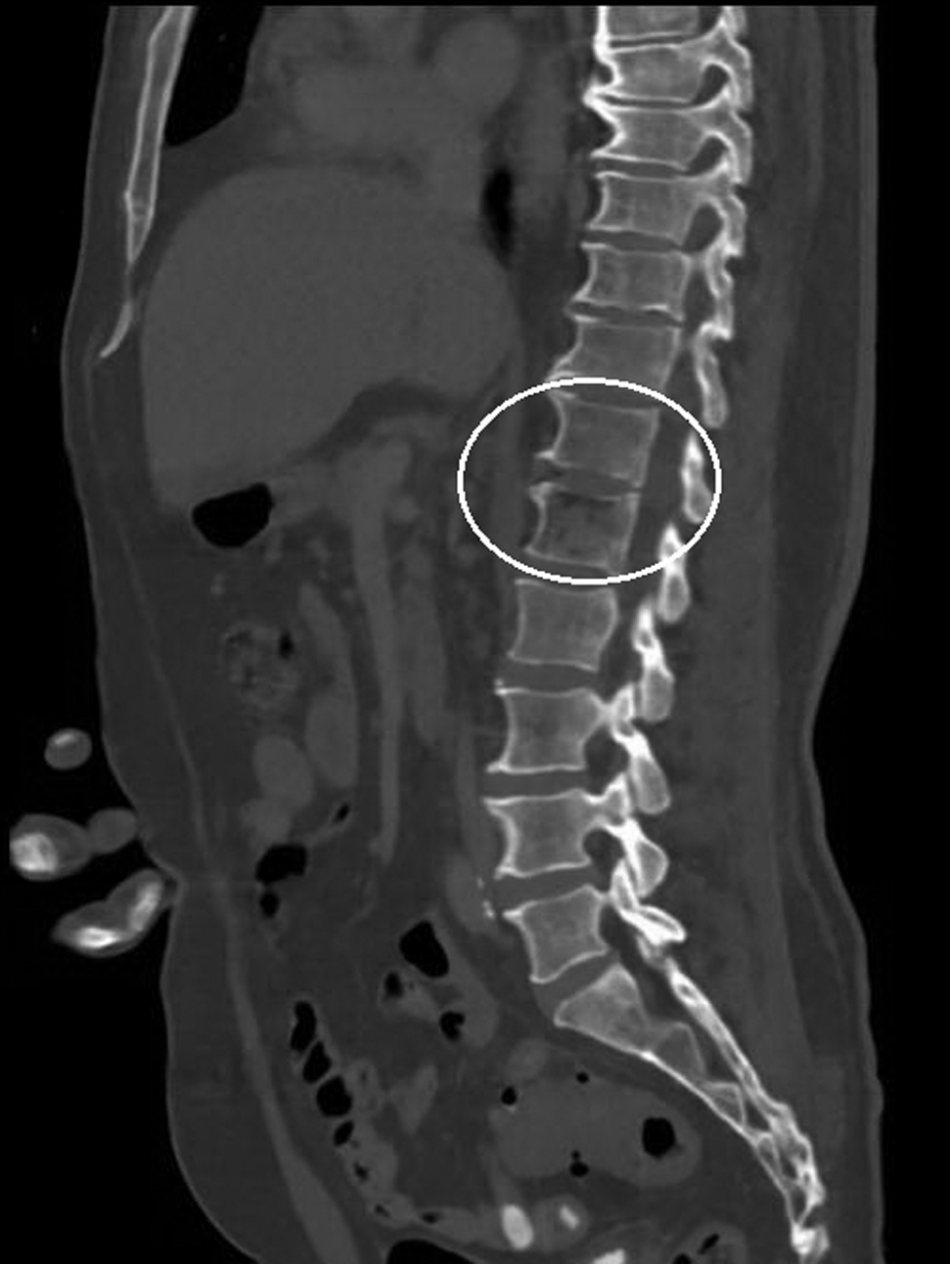
A sagittal bone view of computed tomography of the abdomen and pelvis revealing gas within the twelfth thoracic and first lumbar vertebral body (circle) suspicious for osteomyelitis.

**Image 3.**
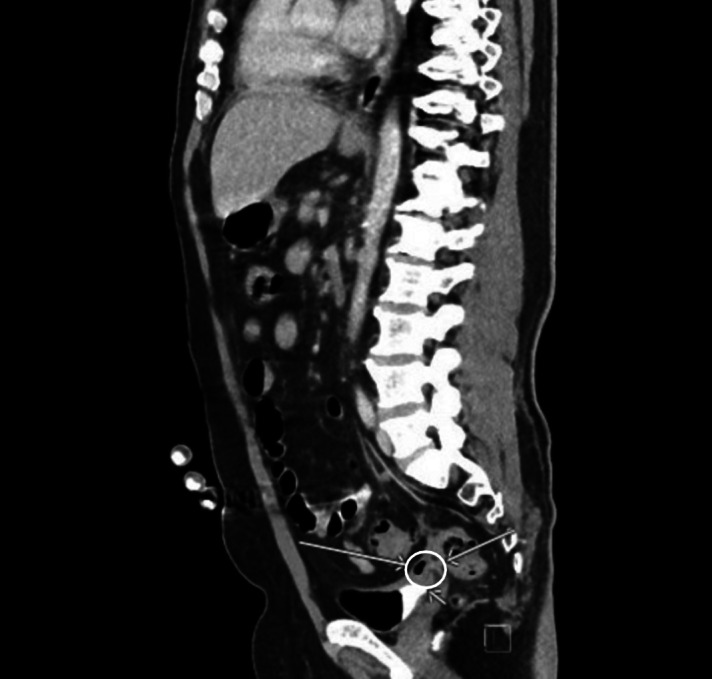
A sagittal view of computed tomography of the abdomen and pelvis demonstrating sigmoid diverticulitis with a colovesicular fistula (circle, arrows).

Initial vital signs were the following: temperature 99.3° Fahrenheit, pulse 120 beats per minute, blood pressure 120/70 millimeters of mercury, respiratory rate 24 breaths per minute, and oxygen saturation 97% on room air. On physical exam, the patient was ill-appearing. There was right clavicle and right-sided chest wall tenderness but no appreciation for crepitus or mass. His cardiopulmonary exam revealed tachycardia. There were no heart murmurs, and lung sounds were clear bilaterally. Laboratory values revealed a white blood count of 7.41 10^9^ per liter (reference range 4.8–11.4 10^9^ per liter), a blood sugar level of 352 milligrams per deciliter (mg/dL) (70–140 mg/dL), and an elevated lactate level of 3.5 millimoles per liter (mMol/L) (0.5–2.0 mMol/L). Additionally, there was an elevated sedimentation rate of 84 millimeters per hour (mm/hr) (0–20 mm/hr), an elevated C-reactive protein level of 22.3 mg/dL (0.0–0.8 mg/dL), and an elevated procalcitonin level of 12.90 micrograms per liter (μg/L) (0.0–0.15 μg/L). A CT of the chest revealed an osseous infection of the manubrium, upper sternum, and right clavicle with an associated mediastinal phlegmon and soft tissue gas extending to the neck and right pectoralis major muscle (Image 1). A CT of the abdomen and pelvis revealed osteomyelitis and discitis of the 12^th^ thoracic and first lumbar vertebral body with associated gas in the psoas muscle body at that level (Image 2). Additionally, sigmoid diverticulitis with colovesicular fistula was noted on the CT abdomen and pelvis (Image 3), mentioned by the patient as a notable finding at the outside hospital.

Differential diagnosis for this patient included septic shock secondary to *Klebsiella pneumoniae* bacteremia, with hematogenous spread to the manubrium and first lumbar vertebral body. The source was suspected to be from the sigmoid diverticulitis with a colovesicular fistula. Alternative differential diagnoses could include necrotizing fasciitis given the presence of gas in the nearby psoas and pectoral muscle, pulmonary embolism, or endocarditis based on fever and chest pain.

The patient was started on broad spectrum antibiotics (piperacillin-tazobactam, vancomycin, and clindamycin) and given a 1,500 milliliter (mL) lactated Ringer’s bolus while in the ED for presumed sepsis. The patient then became hypotensive and was started on a norepinephrine drip with additional crystalloid fluids given. Cardiothoracic surgery was consulted for chest findings. Neurosurgery was consulted for the spine findings, and colorectal surgery was consulted to address the fistula. After being evaluated by cardiothoracic surgery, the patient was immediately taken to the operating room (OR) from the ED for a manubriectomy and for source control. In the OR, the surgeons performed a neck exploration, an incision and drainage, a manubriectomy, and a resection of the right sternoclavicular joint. The patient was then admitted to the surgical intensive care unit after being intubated, and he continued to require vasopressors. Surgical, blood, urine and respiratory cultures grew *K pneumoniae*, which was presumed to be the source of infection. On day six, the patient was found to have additional septic emboli on CT head. A lumbar puncture was performed on day eight; however, cerebrospinal fluid cultures grew no bacteria. The patient was eventually extubated, weaned off vasopressors, and managed on the medical/surgical floor on day 11.

During his hospital stay on the surgical floor, the patient completed a six-week course of intravenous (IV) antibiotics and was switched to oral antibiotics. On hospital day 30, the patient underwent additional surgery for a right sternocleidomastoid muscle flap, debridement of the sternoclavicular joint, and closure of the neck wound. After a 34-day hospital course, the patient was discharged on oral levofloxacin for an additional two weeks with follow-up with cardiothoracic surgery and infectious disease specialists.

## DISCUSSION

Few case reports have been published regarding primary sternal osteomyelitis, with the thought that most stem from hematogenous spread.^4^ ^,^ ^5^ This again is supported by our patient’s presentation, where there were multiple sites of infection including vertebral osteomyelitis, extensive neck and psoas muscle abscesses, and septic emboli infarcts. In hematogenous osteomyelitis, bacteria in the blood flowing through the vasculature in the bone can adhere to it. Bacteria may cause inflammatory changes, breaking down the bony cortex and periosteum, which can cause further invasion of the bacteria and necrosis of the bone.^6^ Typically, risk factors for hematogenous osteomyelitis include endocarditis, IV drug use, sickle cell disease, hemodialysis, orthopedic hardware, and intravascular devices.^7^ However, this patient did not present with any known risk factors. The patient’s history of diabetes mellitus may have been a risk factor for contiguous spread from diabetic ulcers into the adjacent bone. Additionally, the patient presented with only chest pain with no chest mass as seen in other case reports of sternum osteomyelitis.^5^ ^,^ ^8^ ^,^ ^9^ ^,^ ^10^

Furthermore, when taking a closer look at the pathogen isolated in this case, *K pneumoniae* was found both in the vertebrae and the manubrium surgical cultures. *K* *pneumoniae* is a typical anaerobic organism of the gastrointestinal (GI) tract. One study showed it to cause severe bacterial infections where there was a correlation with increased morbidity seen in patients with GI fistulas.^11^ Similarly, with this case it is assumed that the fistula may have caused hematogenous spread of the infection, thus causing the multiple sites of osteomyelitis. Treatment for hematogenous osteomyelitis is normally IV antibiotics alone.^4^ However, for this case a manubriectomy was performed for source control due to the patient’s septic shock. Typically, surgical treatment for sternum osteomyelitis has included surgical debridement, surgical washout, and even hyperbaric oxygen therapy.^5^ ^,^ ^12^ In addition to surgery, this patient did receive a prolonged course of IV and oral antibiotics.

## CONCLUSION

Hematogenous osteomyelitis to the manubrium is rare and may present with only chest pain. Workup includes obtaining inflammatory markers, as well as CT imaging of the chest. Additionally, it is important to consider other sources that may have seeded the manubrium, such as in this patient with colovesicular fistula, and to obtain additional imaging/workup to evaluate for multiple sites of infection. Once the diagnosis is made, treatment at minimum should include IV antibiotics but may require surgical intervention such as debridement with washout or even a manubriectomy.
